# Supplementary material for: Activation of GPR37 in macrophages confers protection against infection-induced sepsis and pain-like behaviour in mice
Source: Nat Commun. 2021 Mar 17;12:1704. doi: 10.1038/s41467-021-21940-8 (PMC7969930; doi:10.1038/s41467-021-21940-8)
Supplement: Supplementary file 1 — Supplementary Information [file 41467_2021_21940_MOESM1_ESM.pdf]

Supplementary information

Activation of GPR37 in macrophages confers protection against infection-induced sepsis and pain-like behaviour in mice

Sangsu Bang[1*], Christopher R Donnelly[1*], Xin Luo[1*], Maria Toro-Moreno[2*], Xueshu Tao[1], Zilong Wang[1], Sharat Chandra[1], Andrey V Bortsov[1], Emily R. Derbyshire[2], and Ru-Rong Ji[1,3,4]

[1]Center for Translational Pain Medicine, Department of Anesthesiology, Duke University Medical Center, Durham, North Carolina, 27710

[2]Department of Chemistry, Duke University, Durham, North Carolina, 27708

[3]Department of Neurobiology, Duke University Medical Center, Durham, North Carolina, 27710

[4]Department of Cell Biology, Duke University Medical Center, Durham, North Carolina, 27710

Email: ru-rong.ji@duke.edu

Table of contents

Supplementary Figures

Supplementary Figure 1.
Supplementary Figure 2.
Supplementary Figure 3.
Supplementary Figure 4.
Supplementary Figure 5.
Supplementary Figure 6.

Supplementary Tables

Supplementary Table 1. A list of FDA-approved natural compounds used for screening
Supplementary Table 2. Antibodies used for flow cytometry studies
Supplementary Table 3. Summary of primers for genotyping $Gpr37^{-/-}$ mice
Supplementary Table 4. Number of animals used across experiments
Supplementary Table 5. Summary of statistical tests in main and supplementary figures

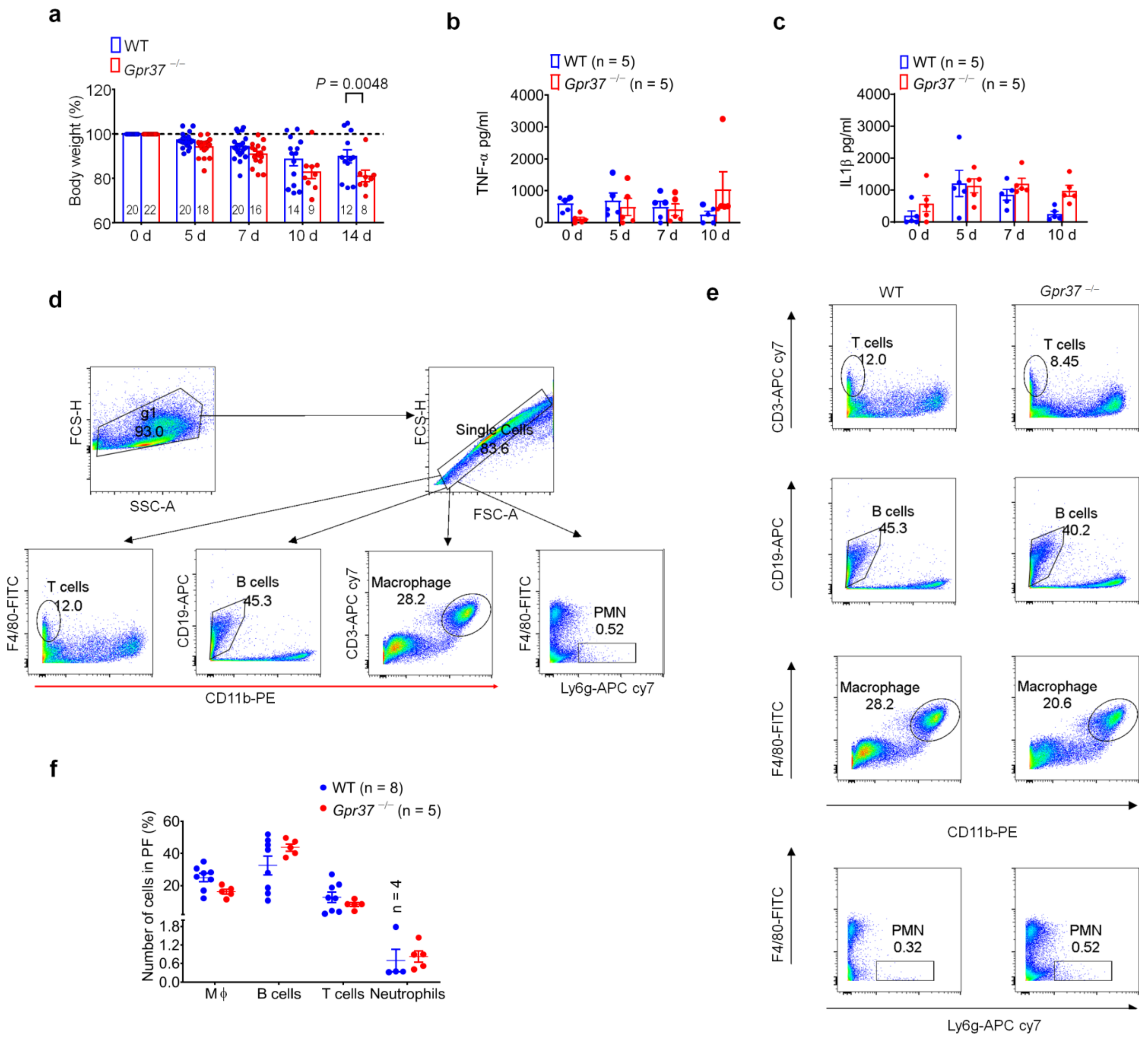

Supplementary Figure 1. (a) Weight change (in %) following inoculation of *P.b.* (1 x 10^4 sporozoites, I.V.) in WT and *Gpr37* $^{-/-}$ mice. Sample sizes are indicated within each column. Changes in weight were only observed at later stages of infection. Note that measurements were only performed on surviving mice at each time point. **(b-c)** Time course of serum TNF-α (b) and IL-1β (c) after *P.b.* inoculation of WT and *Gpr37*$^{-/-}$ mice (n = 5/group). (**d**) Gating strategy for base line immune cell population and PMN calculation at peritoneal cavity, liver, or spleen in WT and *Gpr37* $^{-/-}$ mice. 1 x10^6 cells were collected from each mouse. (**e**) Representative images for B cells, macrophage cells and neutrophils. (**f**) Quantification of peritoneal immune cell population in WT (n = 4-8) and *Gpr37*$^{-/-}$ mice (n = 5). Data are expressed as the mean ± s.e.m. and were statistically analyzed by Two-Way ANOVA with Bonferroni's post-hoc test.

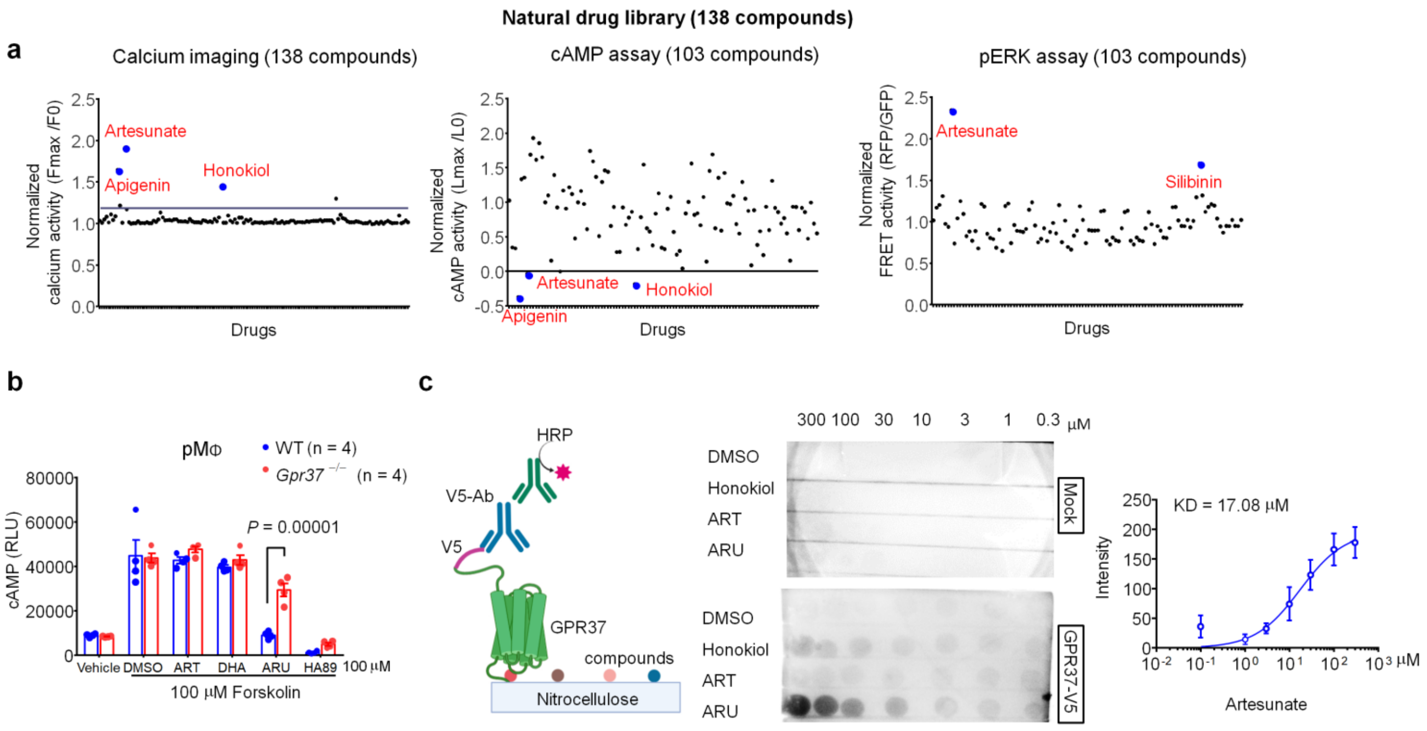

Supplementary Figure 2. (a) library of 138 FDA-approved natural compounds (**Supplementary table 1**) was screened for calcium activity using GCaMP6f (left), cAMP activity using a cAMP-BRET assay (middle), or pERK activity using a pERK-FRET assay (right) in HEK293T cells expressing GCaMP6f and hGPR37. All compounds were used at 10 µM for 20 min treatment. Compounds labeled in red exhibited profiles consistent with activity as a potential GPR37 agonist. **(b)** cAMP levels were measured in pMΦ from WT and *Gpr37*$^{-/-}$ mice treated with forskolin (100 µM) in conjunction with DMSO (negative control), artemisinin (ART), Dihydroartemisinin (DHA), artesunate, or H89 (PKA inhibitor as positive control), *n* = 4 mice/group. Data are expressed as the mean ± s.e.m. and were statistically analyzed by Two-Way ANOVA with Bonferroni's post-hoc test. (**c**) Left: schematic for overlay binding assay. Putative GPR37 agonists were immobilized on a nitrocellulose membrane followed by incubation with lysate of V5-tagged hGPR37 transfected HEK293 cells. Middle: representative dot blot image showing GPR37 binding to DMSO, Artemisinin (ART), Honokiol, and Artesunate (ARU). Right: Quantification of intensity for artesunate. These experiments were repeated 3 times with similar results. Data are expressed as the mean ± s.e.m. and were statistically analyzed using Two-Way ANOVA with Bonferroni's post-hoc test (b).

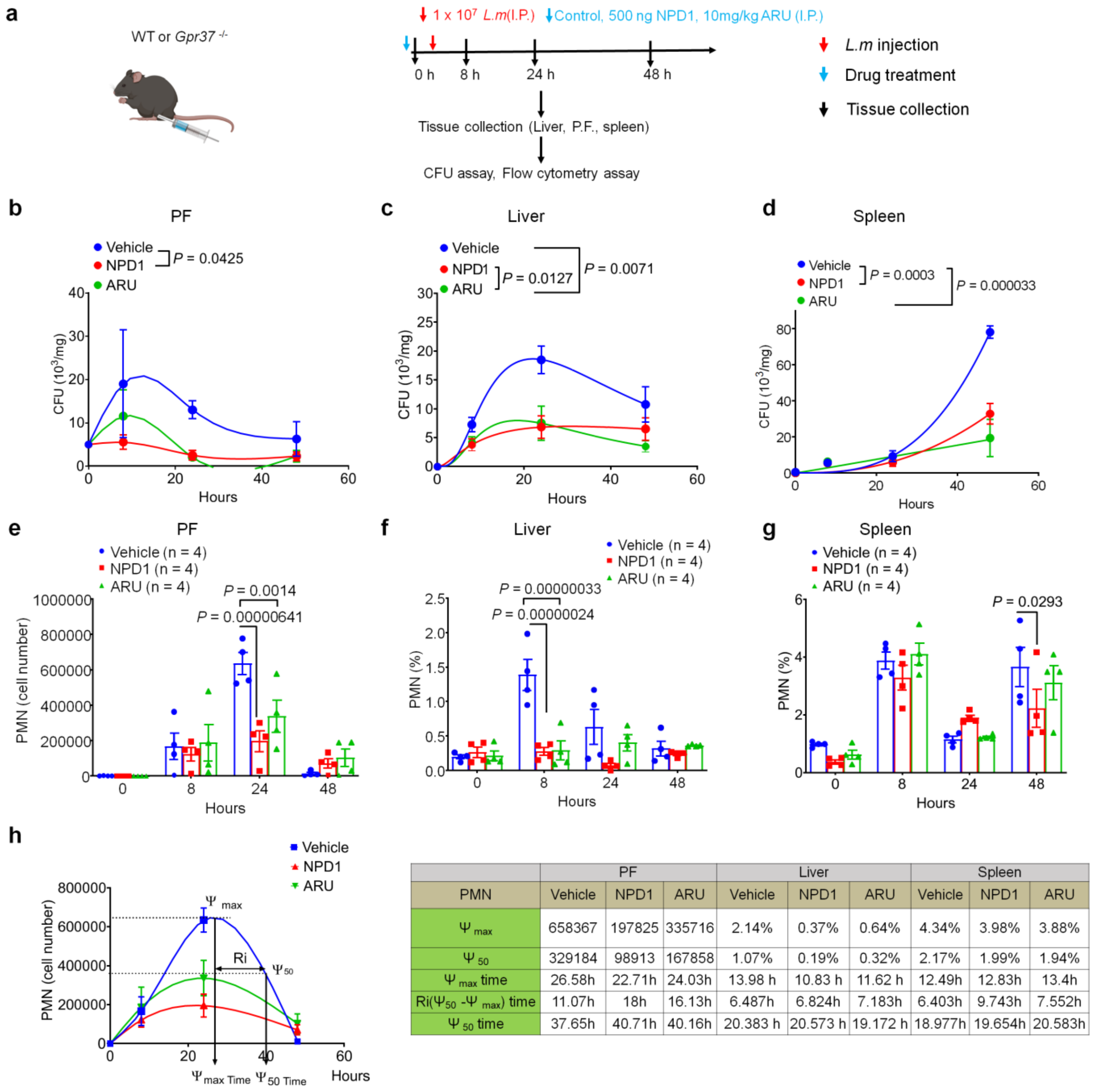

	PF			Liver			Spleen		
PMN	Vehicle	NPD1	ARU	Vehicle	NPD1	ARU	Vehicle	NPD1	ARU
Ψ_{max}	658367	197825	335716	2.14%	0.37%	0.64%	4.34%	3.98%	3.88%
Ψ_{50}	329184	98913	167858	1.07%	0.19%	0.32%	2.17%	1.99%	1.94%
Ψ_{max} time	26.58h	22.71h	24.03h	13.98 h	10.83 h	11.62 h	12.49h	12.83h	13.4h
Ri(Ψ_{50} -Ψ_{max}) time	11.07h	18h	16.13h	6.487h	6.824h	7.183h	6.403h	9.743h	7.552h
Ψ_{50} time	37.65h	40.71h	40.16h	20.383 h	20.573 h	19.172 h	18.977h	19.654h	20.583h

Supplementary Figure 3. (a) Experimental design for calculation of measuring of PMN or bacterial load in *L.m.* infection with vehicle (DMSO), ARU (10 mg/kg), or NPD1 (500 ng) into WT mice (1 x 10^7 *L.m.*, I.P.). **(b-d)** Time dependent tissue *L.m.* level using colony formation unit assay (CFU) after 1 x 10^7 CFU infection (I.P.) with vehicle, 500 ng NPD1 or 10 mg/kg ARU in WT mice (I.P.; *n* = 4 mice each time point and groups). The tissue bacterial load was measured in peritoneal cavity (a), liver (b), and spleen (c) after 24 hours of *L.m.* infection. **(e-g)** Time dependent PMN infiltration level using flow cytometry analysis after 1 x 10^7 CFU *L.m.* infection with vehicle, 500 ng NPD1 or 10 mg/kg ARU in WT mice (I.P.; *n* = 4 mice each time point and groups). The tissue PMN level were calculated by number of Ly6g-APC cy7 positive cells in 10 folded diluted peritoneal fluid (e) or % of ly6g positive cells in liver (f) or spleen (g) each time point (see gating strategy in supplementary Fig 1d). **(h)** Example calculation of PMN kinetics (left). Calculating the PMN kinetics value for maximum infiltration level and time, or resolution time (Ri) at peritoneal fluid after *L.m.* infection with vehicle, NPD1, or ARU in WT mice using gaussian equation in P.F., liver, and spleen (right). Data are expressed as the mean ± s.e.m. and were statistically analyzed using Two-Way ANOVA with Tukey's post-hoc test (b-g).

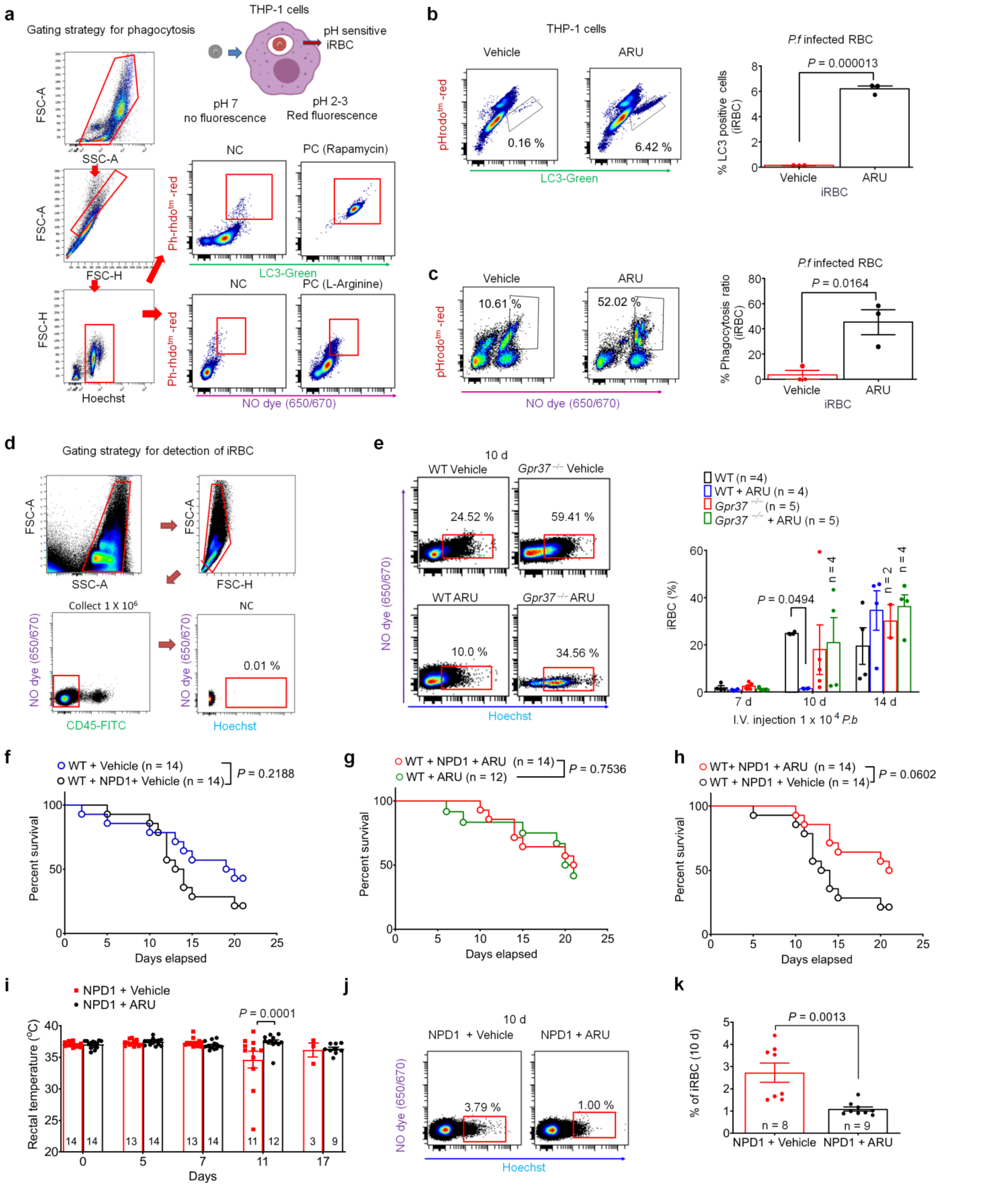

Supplementary Figure 4. **(a)** Gating strategy for detection of THP-1 phagocytosis of *Plasmodium falciparum* (*P.f.*)-infected RBC (iRBC) and pMϕ phagocytosis of *P.b.*-infected RBCs (iRBCs) for fig 4h-k. (**b-c**) Flow cytometry analysis of THP-1 phagocytosis of *P.f.*-infected RBC (10^6 cells) phagocytosis following treatment with

vehicle or ARU (30 µM, 1 hour). Analysis of phagocytosis by flow cytometry was performed using the pH sensitive dye pHrodo and either a LC3 indicating dye (a marker of autophagosome formation) or NO dye (650/670). **b** Left: representative images of flow cytometry results for pHrodo$^+$/LC3$^+$ THP-1 cells following co-culture with *P.f.*-infected RBCs. The proportion of pHrodo$^+$/LC3$^+$ THP-1 cells is indicated. Right: quantification of flow cytometry results (10^6 THP-1 cells analyzed per group from n = 3 experimental repeats). **c** Left: representative images of flow cytometry for pHrodo$^+$/NO$^+$ THP-1 cells following co-culture with *P.f.*-infected RBCs. The proportion of pHrodo$^+$/NO$^+$ THP-1 cells is indicated. Right: quantification of flow cytometry results (10^6 THP-1 cells analyzed per group from n = 3 experimental repeats). (**d**) Gating strategy for detection of *P.b.*-infected RBC in peripheral blood samples using untreated WT mice as a negative control. iRBCs are identified as NOS$^-$ CD45$^-$ NO$^-$ Hoechst$^+$ cells. (**e**) Left: representative images of peripheral blood samples from WT or *Gpr37*$^{-/-}$ mice following treatment with vehicle or ARU 10d after *P.b.* inoculation analyzed by flow cytometry. The proportion of iRBCs (NO$^-$/Hoechst$^+$) for each representative image is indicated. Left: quantification of the proportion of iRBCs in each sample 10^6 of RBC analyzed per sample, n = 5 mice/group. (**f-h)** WT mice were administered *P.b.* (1 x 10^4 sporozoites, I.V.) followed by 500 ng NPD1 or 500 ng NPD1 + 10 mg/kg ARU (3 times) (I.P.) **f** Survival curve was replotted with WT-Vehicle group (Fig. 5j) vs. WT-NPD1+Vehicle. **g** Survival curve was replotted with WT-ARU group (Fig. 5j) vs. WT-NPD1+ARU. **h** survival curve for WT-NPD1+vehicle vs. WT-NPD1+ARU. (**i**) Rectal temperature at the indicated timepoints of mice in the treatment groups outlined in Fig. 5i (n = 14 mice/group). (**j**) Representative images of peripheral blood samples from WT mice following treatment with NPD1 or NPD1 + ARU 10 d after *P.b.* inoculation analyzed by flow cytometry. The proportion of iRBCs (NO$^-$/Hoechst$^+$) for each representative image is indicated (see gating strategy in supplementary Fig 4d). (**k**) Quantification of the proportion of iRBCs in each sample 10^6 of RBC analyzed per sample, n = 8 or 9 mice/group. Data are expressed as the mean ± s.e.m. and were statistically analyzed using Mantel-Cox test (f-h), Two-Way ANOVA with Tukey's post-hoc test (e) or Bonferroni test (i) or unpaired two tailed t-test (b, c, k).

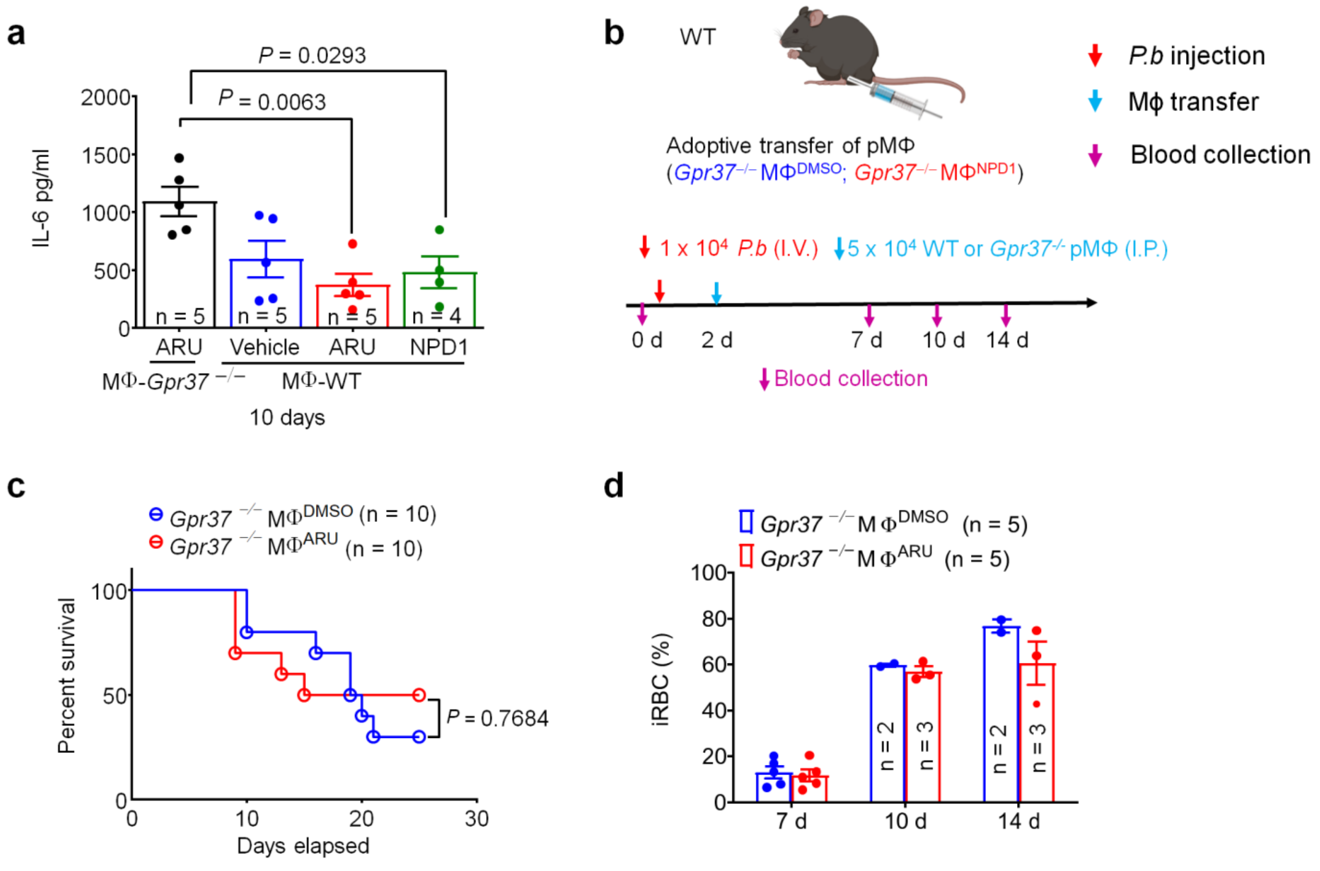

Supplementary Figure 5. (a) Serum IL-6 levels 10 d after inoculation with *P.b.* in mice receiving adoptive transfer of MΦ from WT or *Gpr37*$^{-/-}$ mice primed with vehicle, ARU (30 µM), or NPD1 (30 nM) (24 hours; 37 ℃). **(b)** Experimental design for adoptive transfer of *Gpr37*$^{-/-}$ macrophages primed with vehicle (DMSO) or ARU (30 µM, 24 hours, 37 ℃) into WT mice 2 d following inoculation with *P.b.* (1 x 10^4 sporozoites, I.V.). **(c)** Survival curves of the mice described in panel (n = 10). **(d)** Quantification of the proportion of iRBCs from peripheral blood samples, measured by flow cytometry analysis (Hoechst$^+$/CD45$^-$) 7 d, 10 d, or 14 d after inoculation (n = 2-5 mice/group, as samples were limited at later stages by infection-induced mortality). See gating strategy in supplementary Fig 4d). Data are expressed as the mean ± s.e.m. and were statistically analyzed using One-Way ANOVA with Tukey's post-hoc test (a), or statistically analyzed by Mantel-Cox test (c).

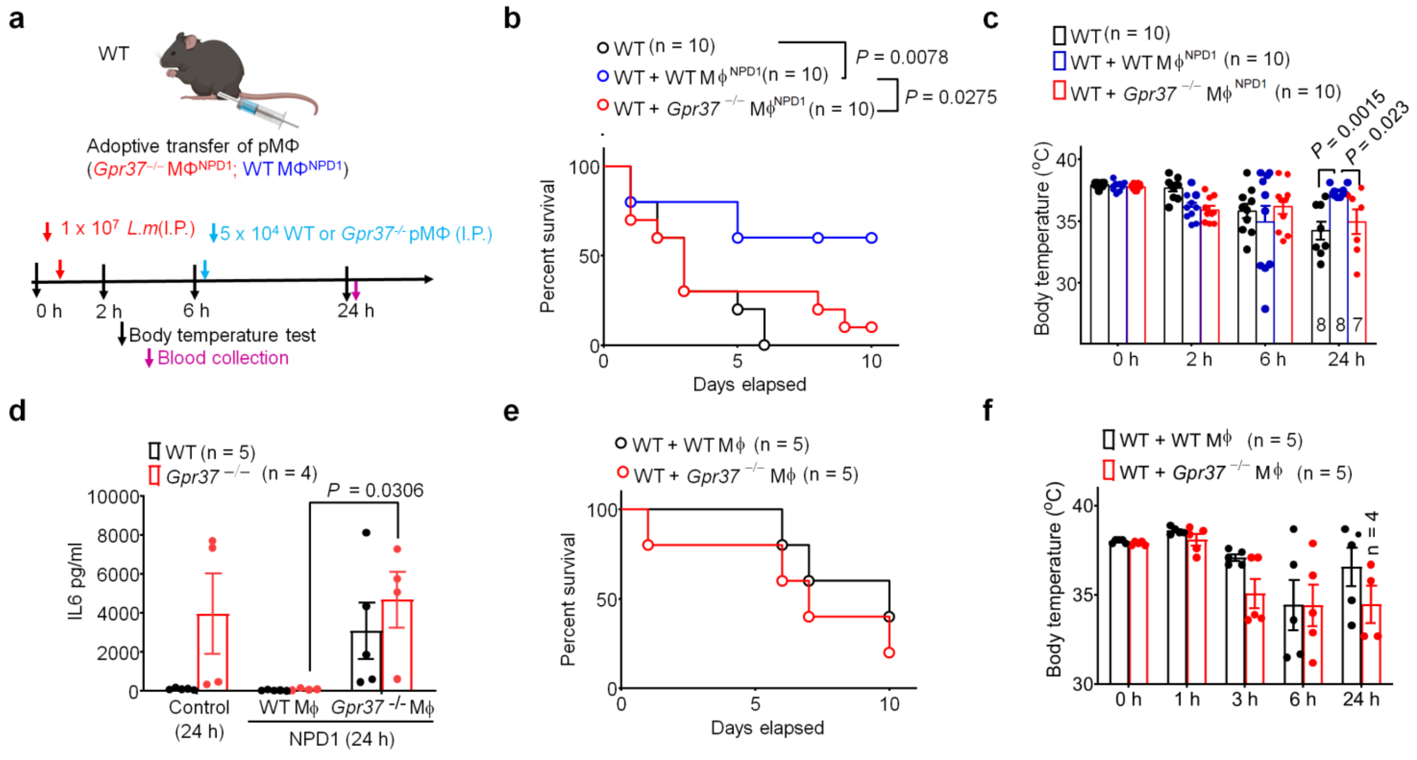

Supplementary Figure 6. **(a)** Experimental design to test whether NPD1 priming of pMΦ alters the time course or severity of *L.m.*-induced infection and septic death. **(b-d)** WT mice were administered *L.m.* (1 x 10^7, I.P.) followed by adoptive transfer of NPD1-primed pMΦ from WT or *Gpr37*$^{-/-}$ mice 6 h later (5 x 10^4 cells, I.P.) **(b)** Survival curves and **(c)** rectal temperature at the indicated timepoints of mice in the treatment groups outlined in a (*n* = 10 mice/group). **(d)** Serum IL-6 levels in each group 24 h after *L.m.* inoculation (18 h after adoptive transfer; *n* = 5 for WT *n* = 4 for *Gpr37*$^{-/-}$ mice). **(e-f)** WT mice were administered *L.m.* as in a, followed by adoptive transfer of vehicle-primed WT or *Gpr37*$^{-/-}$ pMΦ 6h later (5 x 10^4 cells, I.P.). **(e)** Survival curves and **(f)** rectal temperature at the indicated time points for each treatment group (*n* = 5 mice/group). Data are expressed as the mean ± s.e.m. and were statistically analyzed using Mantel-Cox test (b, e) or Two-Way ANOVA with Tukey's post-hoc test (c, d), Bonferroni's post-hoc test (f).

Supplementary Table 1: A list of FDA-approved natural compounds used for screening.

aesculin	bilobalide	epigallocatechin gallate	hypoxanthine	myricetin	Polydatin	sophocarpine
Aloin	biochanin	ergosterol	icariin	myricitrin	puerarin	stigmasterol
AMGZ	caffeic acid	fisetin	indole carbinol	nalidixic acid	quercetin	syneohrine
ampelopin	chlorogenic acid	fomononectin	indolebutyric acid	naringenin	Quercetin	tangeretin
amygdalin	chrysophenol	fumalic acid	indrubin	naringin	rhamonose	tanshinone
Anamorelin	coQ10	gastrodine	inosine	Naringin	rhenin	tashinone
Ampiroxicam	cryptotanshinone	gossypol	iprifvone	neohesperidin	rutaecarpine	taxifolin
andrographolid	curcumol	gramine	isoliguirigenin	neohesperidin	rutin	tetrahydropapaverine
apigenin	cyclosporinA	guanosine	kaempferol	nobiletin	rutundin	tettradrine
apocynin	cyclpcytidine	gynostemma	kinetin	oeanolic acid	salicin	theobrimide
Arbutin	cytisine	HDCA	lappaconnie	oridonin	salidroside	triptolide
artesunate	danshensu	hematoxylin	l-carnitine	orotic acid	sciareolide	troxerutin
asiatic acid	DHA	hesperetin	limonin	oxymatrine	sclareol	urosolic
astragaloside	dioscine	hesperidin	luteolin	paenol	scopolamine	usniain
azomycin	diosemetin	honokiol	magnolol	palmatine	seasamin	vanilin
baicalein	diosgenin	hordenin	matrine	parthenolide	shikimic	vanillyacetate
baicalin	diosmin	HTP	menensin	phloretin	silicinin	xanthone
bebuxine	dl carnitine	huperizine a	methylhesperidin	phlorizin	silymarin	yohimbine
berberine	emodin	hydrocyecdyone	methylumbelliferone	physcione	sinomenine	
bergenin	enoxolone	hydroxycamptothecin	morin hydrate	piperin	sitosterol	

Supplementary Table 2: Antibodies used for flow cytometry studies.

laser	405	488	488	633	633	Tissue	Application
filter	450/50	530/30	570/20	660/20	780/60		
RBC FACS	Hoechst dye	CD45-FITC	pHrodo dye	NOS dye		blood	detection of iRBC
dilution	1 µg/ml	1:100	1 µg/ml	1:50			
pMΦ phagocytosis	Hoechst dye	LC3-488	pHrodo dye	NOS dye		pMΦ	Macrophage phagocytosis
dilution	1 µg/ml	1 µg/ml	1 µg/ml	1:50			
THP1 phagocytosis	Hoechst dye	LC3-488	pHrodo dye	NOS dye		THP1	
dilution	1 µg/ml	1 µg/ml	1 µg/ml	1:50			
pMΦ elimination & PMN level	Hoechst dye	F4/80-FITC		CD11b-APC	Ly6g- APC Cy7	Peritoneal fluid	
dilution	1 µg/ml	1:100		1:100	1:100		
PMN		F4/80-FITC	CD11b-PE	CD19-APC	Ly6g-APC cy7	Liver, spleen, Peritoneal	Listeria induced Infiltration
dilution		1:100	1:100	1:100	1:100		
Base line cells		F4/80-FITC	CD11b-PE	CD19-APC	CD3-APC cy7	Peritoneal fluid	
dilution		1:100	1:100	1:100	1:100		

Antibody information

Antibody	Catalog#	company	isotype	application
cd16/32	101302	Biolegend	Mouse IgG1, κ	Blocking
F4/80-FITC	123107	Biolegend	Rat IgG2a, κ	FACS
CD11b-APC	101211	Biolegend	Rat IgG2a, κ	FACS
CD45 (30-F11), FITC,	11-0451-82	eBioscience	Rat IgG2b, k	FACS
Ly6g APC cy7	127623	Biolegend	Rat IgG2a, k	FACS
CD11b-PE	101207	Biolegend	Rat IgG2a, κ	FACS
CD19-APC	115511	Biolegend	Rat IgG2a, κ	FACS
CD3-APC cy7	100221	Biolegend	Rat IgG2a, κ	FACS

Supplementary Table 3: Summary of primers for genotyping *Gpr37*$^{-/-}$ mice.

Primer name	Sequence 5' -> 3'
moIMR0012	GGG TGG GAT TAG ATA AAT GCC TGC TCT
oIMR5111	AAC GGG TCT GCA GAT GAC TGG GTT
oIMR5112	GGC CAA GAG AGA ATT GGA GAT GCT C
WT product: 237 base pairs.<br>Gpr37 KO product: 400 base pairs.<br>For protocol, see:<br>https://www.jax.org/Protocol/UrlAsPDF?stockNumber=005806&protocolID=24813	

Supplementary Table 4: Number of animals used across experiments.

Figures		Sample size	Number of groups	Number of animals
Fig1	Fig.1b	n = 14-15 mice	4	30 WT mice (15 M + 15 F), 28 *Gpr37*$^{-/-}$ mice (15 M + 13 F)
	Fig. 1c	n = 14-15 mice	4	30 WT mice (15 M + 15 F), 28 *Gpr37*$^{-/-}$ mice (15M + 13 F), same mice with Fig. 1b
	Fig. 1d	n = 4 samples	4	8 WT mice (4 M + 4F), 8 *Gpr37*$^{-/-}$ mice (4 M + 4 F), same mice with Fig. 1b
	Fig. 1f	n = 14-17 mice	4	33 WT mice (16 M + 17 F), 30 *Gpr37*$^{-/-}$ mice (15 M + 15 F)
	Fig. 1g	n = 6-8 mice	4	15 WT mice (7M + 8F), 12 *Gpr37*$^{-/-}$ mice (6 M + 6 F), same mice with Fig. 1f
	Fig. 1h	n = 6 samples	4	12 WT mice (6M + 6F), 12 *Gpr37*$^{-/-}$ mice (6 M + 6 F), same mice with Fig. 1f
	Fig. 1i	n = 5-6 samples	12	6 WT mice (3 M + 3 F), 5 *Gpr37*$^{-/-}$ mice (3M + 2 F)
	Fig. 1j	n = 3 samples	12	3 WT mice (2 M + 1 F), 3 *Gpr37*$^{-/-}$ mice (2 M + 1 F), same mice with Fig. 1i
	Fig. 1k	n = 5 samples	12	5 WT mice (3 M + 2 F), 5 *Gpr37*$^{-/-}$ mice (3 M + 2 F), same mice with Fig. 1i, j
Fig 2	Fig. 2b	n = 20-22 mice	2	20 WT mice (10 M + 10 F), 22 *Gpr37*$^{-/-}$ mice (12 M + x 10 F)
	Fig. 2c	n = 20-22 mice	2	20 WT mice (10 M + 10 F), 22 *Gpr37*$^{-/-}$ mice (12 M + 10 F), same mice with Fig. 2b
	Fig. 2d	n = 5 samples	2	5 WT mice (3M + 2 F), 5 *Gpr37*$^{-/-}$ mice (3 M + 2F), same mice with Fig. 2b
	Fig. 2e	n = 5 samples	2	5 WT mice (3 M + 2 F), 5 *Gpr37*$^{-/-}$ mice (3 M + 2 F), same mice with Fig. 2b
	Fig. 2f	n = 13 samples	2	13 WT mice (7 M + 6 F), 13 *Gpr37*$^{-/-}$ mice (7 M + 6 F), same mice with Fig. 2b
Fig 4	Fig. 4b	n = 5 cultures	4	5 WT mice (3 M + 2 F), 5 *Gpr37*$^{-/-}$ mice (3 M + 2 F)
	Fig. 4d	n = 5 cultures	4	5 WT mice (3 M + 2 F), 5 *Gpr37*$^{-/-}$ mice (3 M + 2 F)
	Fig 4f	n = 4~6 mice	6	16 WT mice(8 M + 8 F), 16 *Gpr37*$^{-/-}$ mice (8 M + 8 F)
	Fig 4g	n = 6~12 sample	6	12 WT mice(6 M + 6 F), 12 *Gpr37*$^{-/-}$ mice (6 M + 6 F)
	Fig. 4i	n = 3 cultures	4	3 WT mice (2 M + 1 F), 3 *Gpr37*$^{-/-}$ mice (2 M + 1 F)
	Fig. 4k	n = 3 cultures	4	3 WT mice (2 M + 1 F), 3 *Gpr37*$^{-/-}$ mice (2 M + 1 F) ,same mice with Fig. 4i
Fig 5	Fig. 5b	n = 15 mice	4	30 WT mice (15 M + 15 F), 30 *Gpr37*$^{-/-}$ mice (15 M + 15 F)
	Fig. 5c	n = 15 mice	4	30 WT mice (15M + 15 F), 30 *Gpr37*$^{-/-}$ mice (15 M + 15F), same mice with Fig. 5b
	Fig. 5d	n = 4 samples	4	8 WT mice (4 M + 4 F), 8 *Gpr37*$^{-/-}$ mice (4 M + 4F), same mice with Fig. 5b
	Fig. 5f	n = 8 mice	4	16 WT mice (8 M + 8F), 16 *Gpr37*$^{-/-}$ mice (8 M + 8 F)
	Fig. 5g	n = 8 mice	4	16 WT mice (8M + 8 F), 16 *Gpr37*$^{-/-}$ mice (8 M + 8 F), same mice with Fig. 5f
	Fig. 5h	n = 4 samples	4	8 WT mice (4 M + 4 F), 8 *Gpr37*$^{-/-}$ mice (4 M + 4F), same mice with Fig. 5f
	Fig. 5j	n = 8-14 mice	4	26 WT mice (13 M + 13 F), 16 *Gpr37*$^{-/-}$ mice (8 M + 8 F)
	Fig. 5k	n = 8-12 mice	4	21 WT mice (10 M + 11 F), 16 *Gpr37*$^{-/-}$ mice (8 M + 8 F), same mice with Fig. 5j
	Fig. 5l	n = 8 samples	4	16 WT mice (8 M + 8 F), 16 *Gpr37*$^{-/-}$ mice (8 M + 8 F), same mice with Fig. 5j and add 4 Gpr37$^{-/-}$ (4 M)
Fig 6	Fig. 6b	n = 18-20 mice	2	38 WT mice (20 M + 18 F)
	Fig. 6c	n = 18-20 mice	2	38 WT mice (20 M + 18 F), same mice with Fig. 6b
	Fig. 6d	n = 6 samples	2	24 WT mice (12 M + 12 F), same mice with Fig. 6b
	Fig. 6f	n = 6-8 samples	2	14 WT mice (7 M + 7 F), same mice with Fig. 6b
	Fig. 6h	n = 3 samples	2	6 WT mice (3 M + 3 F)
Fig 7	Fig. 7b	n = 6-16 mice	4	46 *Gpr37*$^{-/-}$ mice (25 M + 21 F) ,4 WT mice(2 M + 2 F), 2 *Gpr37*$^{-/-}$ mice (1 M + 1 F) for transfer
	Fig. 7c	n = 6-9 mice	4	32 *Gpr37*$^{-/-}$ mice (16 M + 16 F) same mice with Fig. 6B
	Fig. 7f	n = 5-10 mice	4	35 *Gpr37*$^{-/-}$ mice (20M + 15 F), 4 WT mice(2 M + 2 F), 2 *Gpr37*$^{-/-}$ mice (1 M + 1 F) for transfer
	Fig. 7g	n = 5-10 mice	4	35 *Gpr37*$^{-/-}$ mice (20 M + 15 F) same mice with Fig. 6F
Fig 8	Fig. 8c	n = 5-9 mice	2	5 WT mice (3 M + 2 F), 5 *Gpr37*$^{-/-}$ mice (3 M + 2 F)
	Fig. 8d	n = 14-15mice	2	15 WT mice (7 M + 8 F), 14 *Gpr37*$^{-/-}$ mice (7 M + 7 F)
	Fig. 8e	n = 5 mice	2	5 WT mice (3M + 2 F), 5 *Gpr37*$^{-/-}$ mice (3 M + 2F) same mice with Fig. 8b
	Fig. 8f	n = 5 mice	2	5 WT mice (3 M + 2 F), 5 *Gpr37*$^{-/-}$ mice (3 M + 2 F) same mice with Fig. 5j
	Fig. 8h	n = 9 mice	2	18 *Gpr37*$^{-/-}$ mice (9 M + 9 F), 4 WT mice(2 M + 2 F), 2 *Gpr37*$^{-/-}$ mice (1 M + 1 F) for transfer
	Fig. 8i	n = 9-10 mice	2	10 WT mice (5 M + 5 F), 9 *Gpr37*$^{-/-}$ mice (4 M + 5 F)
sFig 1	sFig. 1a	n = 20-22 mice	2	20 WT mice (10 M + 10 F), 22 *Gpr37*$^{-/-}$ mice (12 M + 10 F), same mice with Fig. 2b
	sFig. 1b	n = 5 mice	2	5 WT mice (3 M + 2 F), 5 *Gpr37*$^{-/-}$ mice (3 M + 2F), same mice with Fig. 2b
	sFig. 1c	n = 5 mice	2	5 WT mice (3 M + 2 F), 5 *Gpr37*$^{-/-}$ mice (3 M + 2 F), same mice with Fig. 2b
	sFig 1d-f	n = 5~8 mice	2	8 WT (4 M + 4 F), 5 *Gpr37*$^{-/-}$ mice (2 M + 3 F)
sFig 2	sFig. 2b	n = 4 mice	2	4 WT (2 M + 2 F), 4 *Gpr37*$^{-/-}$ mice (2 M + 2 F)
sFig3	sFig 3b-d	n = 4 mice	10	52 WT(26 M + 26 F)
	sfFig 3e-g	n = 4 mice	10	48 WT(24 M + 24 F), same mice with sFig3b
sFig 4	sFig. 4d,e	n = 5 samples	4	8 WT mice (4 M + 4 F), 10 *Gpr37*$^{-/-}$ mice (5 M + 5 F), same mice with Fig. 5k
	sFig. 4b	n = 4 cultures	12	2 WT mice (1 M + 1 F), 2 *Gpr37*$^{-/-}$ mice (1 M + 1 F)
	sFig 4f-k	n = 5-14 mice	3	33 WT (17 M + 16 F)
sFig 5	sFig. 5a	n = 4-5 samples	4	19 *Gpr37*$^{-/-}$ mice (10 M + 9 F), same mice with Fig. 7b.
	sFig. 5c	n = 10 mice	2	20 WT mice (10 M + 10 F), 2 *Gpr37*$^{-/-}$ mice (1 M + 1 F) and 2 WT mice(1 M + 1F) for transfer
	sFig. 5d	n = 5 mice	2	5 WT mice (5 M), 5 *Gpr37*$^{-/-}$ mice (5 M), same mice with sFig. 5c
sFig 6	sFig. 6b	n = 10 mice	3	30 WT mice (15 M + 15 F), 2 *Gpr37*$^{-/-}$ mice (1 M + 1 F) and 2 WT mice(1 M + 1F) for transfer
	sFig. 6c	n = 10 mice	3	30 WT mice (15 M + 15 F), same mice with sFig. 6b
	sFig. 6d	n = 4-5 mice	6	15 WT mice (7M + 8F) , 4 *Gpr37*$^{-/-}$ mice (4 M + 4F) ,same mice with Fig. 7f and sFig. 6b
	sFig. 6e	n = 5 mice	2	10 WT mice (10 M) for culture WT mice (1 M), *Gpr37*$^{-/-}$ mice (1 M)
	sFig. 6f	n = 5 mice	2	10 WT mice (10 M), same mice with sFig.6e
**Total number of mice**				452 WT mice (234 M + 218 F), 341 *Gpr37*$^{-/-}$ mice (181 M + 160 F)

Supplementary Table 5: Summary of statistical tests in main and supplementary figures.

Figures	Statistic tests		F/Chi square values	P value	Post-hoc test P values
fig1b	Log-rank (Mantel-Cox) test	Chi square	8.152	P = 0.0037	WT vs WT+NPD1 P=0.0043<br>WT+NPD1 vs Gpr37 KO + NPD1 P=0.008
fig1c	Two-way ANOVA	Tukey's multiple comparisons test	F (3, 147) = 4.410	P = 0.0053	WT vs WT+NPD1 P=0.0019
			F (2, 147) = 132.2	P < 0.0001	WT+NPD1 vs Gpr37 KO+NPD1 P=0.0002
fig1d	Two-way ANOVA	Tukey's multiple comparisons test	F (3, 24) = 4.142	P=0.0168	WT vs WT+NPD1 P=0.0022
			F (1, 24) = 487.5	P<0.0001	WT+NPD1 vs Gpr37 KO+NPD1 P=0.0029
fig1f	Log-rank (Mantel-Cox) test	Chi square	15.83	P<0.0001	WT vs WT+NPD1 P=0.0094<br>WT+NPD1 vs Gpr37 KO + NPD1 P=0.000069<br>WT vs Gpr37 KO P=0.0396
fig1g	Two-way ANOVA	Tukey's multiple comparisons test	F (3, 64) = 6.487	P=0.0007	WT vs WT+NPD1 P=0.0433
			F (2, 64) = 37.70	P<0.0001	WT+NPD1 vs Gpr37 KO+NPD1 P=0.000011
fig1h	Two-way ANOVA	Tukey's multiple comparisons test	F (3, 40) = 10.82	P<0.0001	WT vs WT+NPD1 P=0.0255
			F (1, 40) = 97.32	P<0.0001	WT+NPD1 vs Gpr37 KO+NPD1 P=0.0000001
fig1i	Two-way ANOVA	Bonferroni's multiple comparisons test	F (1, 52) = 1.321	P=0.2556	WT vs WT+NPD1 P=0.0275(LPS)
		Bonferroni's multiple comparisons test	F (5, 52) = 7.438	P<0.0001	WT+NPD1 vs Gpr37 KO+NPD1 P=0.0214(LPS)
fig1j	Two-way ANOVA	Bonferroni's multiple comparisons test	F (5, 24) = 93.18	P<0.0001	WT vs WT+NPD1 P=0.0023 (L.m)
		Bonferroni's multiple comparisons test	F (1, 24) = 8.379	P=0.0080	WT+NPD1 vs Gpr37 KO+NPD1 P=0.000018(L.m)
fig1k	Two-way ANOVA	Bonferroni's multiple comparisons test	F (5, 48) = 27.09	P<0.0001	WT vs WT+NPD1 P=0.0211(LPS)<br>WT vs WT+NPD1 P=0.00014(L.m)
		Bonferroni's multiple comparisons test	F (1, 48) = 3.092	P=0.0850	WT vs Gpr37 KO P=0.005 (LPS)<br>WT vs Gpr37 KO P=0.0374(L.m)
fig2b	Log-rank (Mantel-Cox) test	Chi square	11.21	P=0.0037	
fig2c	Two-way ANOVA	Bonferroni's multiple comparisons test	F (1, 139) = 104.5	P<0.0001	WT vs Gpr37 KO P=0.0000000002
fig2d	Two-way ANOVA	Bonferroni's multiple comparisons test	F (1, 24) = 10.15	P=0.0040	WT vs Gpr37 KO P=0.000011
fig2e	Two-way ANOVA	Bonferroni's multiple comparisons test	F (2, 22) = 25.61	P<0.0001	WT vs Gpr37 KO P=0.0014
fig2f	Unpaired t test	Two-tailed	t=2.273 df=24	P=0.0323	
fig4b	Two-way ANOVA	Bonferroni's multiple comparisons test	F (1, 16) = 5.531	P=0.0318	WT vs WT+ARU P=0.0005
			F (1, 16) = 6.829	P=0.0188	WT+ARU vs Gpr37 KO+ARU P=0.0008
fig4d	Two-way ANOVA	Bonferroni's multiple comparisons test	F (1, 8) = 54.22	P<0.0001	WT vs WT+ARU P=0.00005
			F (1, 8) = 43.26	P=0.0002	WT+ARU vs Gpr37 KO+ARU P=0.000081
fig4f	Two-way ANOVA	Bonferroni's multiple comparisons test	F (1, 26) = 21.28	P<0.0001	WT vs Gpr37 KO P=0.0301<br>WT vs Gpr37 KO P=0.0003
	One-way ANOVA	Tukey's multiple comparisons test	F (2, 13) = 13.49	P=0.0007	Vehicle vs NPD1 P=0.0027<br>Vehicle vs ARU P=0.002
fig4g	Two-way ANOVA	Tukey's multiple comparisons test	F (5, 252) = 20.60	P<0.0001	WT vs WT+NPD1 P=0.0002<br>WT vs WT+ARU P=0.04<br>WT vs Gpr37 KO P=0.0011
fig4i	Two-way ANOVA	Tukey's multiple comparisons test	F (3, 16) = 7.773	P=0.0020	WT vs WT+ARU P=0.000045
			F (1, 16) = 33.83	P<0.0001	WT+ARU vs Gpr37 KO+ARU P=0.000031
fig4k	Two-way ANOVA	Tukey's multiple comparisons test	F (3, 16) = 8.705	P=0.0012	WT vs WT+ARU P=0.00001
			F (1, 16) = 10.73	P=0.0048	WT+ARU vs Gpr37 KO+ARU P=0.000016
fig5b	Log-rank (Mantel-Cox) test	Chi square	11.65	P=0.0087	WT vs WT+ARU P=0.0516<br>WT+ARU vs Gpr37 KO+ARU P=0.0003
fig5c	Two-way ANOVA	Tukey's multiple comparisons test	F (3, 155) = 4.871	P=0.0029	WT vs WT+ARU P=0.0002
			F (2, 155) = 37.08	P<0.0001	WT+ARU vs Gpr37 KO+ARU P=0.000011
fig5d	Two-way ANOVA	Tukey's multiple comparisons test	F (3, 22) = 3.571	P=0.0304	WT vs WT+ARU P=0.0966
			F (1, 22) = 43.37	P<0.0001	WT+ARU vs Gpr37 KO+ARU P=0.0009
fig5f	Log-rank (Mantel-Cox) test	Chi square	11.03	P=0.0116	WT vs WT+ARU P=0.0145<br>WT+ARU vs Gpr37 KO+ARU P=0.0311
fig5g	Two-way ANOVA	Tukey's multiple comparisons test	F (3, 81) = 4.585	P=0.0051	WT vs WT+ARU P=0.0194
			F (2, 81) = 32.90	P<0.0001	WT+ARU vs Gpr37 KO+ARU P=0.0007
fig5h	Two-way ANOVA	Tukey's multiple comparisons test	F (1, 24) = 12.98	P=0.0014	WT vs WT+ARU P=0.0072
fig5j	Log-rank (Mantel-Cox) test	Chi square	10.07	P=0.018	WT vs Gpr37 KO P=0.0608<br>WT+ARU vs Gpr37 KO+ARU P=0.0212
fig5k	Two-way ANOVA	Tukey's multiple comparisons test	F (3, 91) = 3.903	P = 0.0113	WT vs WT+ARU P=0.0196
fig5l	Two-way ANOVA	Tukey's multiple comparisons test	F (3, 56) = 2.590	P=0.0618	WT vs Gpr37 KO P=0.0218
fig6b	Log-rank (Mantel-Cox) test	Chi square	7.529	P=0.0061	
fig6c	Two-way ANOVA	Tukey's multiple comparisons test	F (1, 99) = 4.565	P = 0.0351	control vs clodronate P=0.000044(10d)
fig6d	Two-way ANOVA	Bonferroni's multiple comparisons test	F (1, 20) = 5.952	P = 0.0241	control vs clodronate P=0.0136(10d)
fig6f	Unpaired t test	Two-tailed	t=2.849, df=12	P=0.0147	control vs clodronate P=0.0147
fig6h	Unpaired t test	Two-tailed	t=4.841, df=4	P=0.0084	control vs clodronate P=0.0084
fig7b	Log-rank (Mantel-Cox) test	Chi square	11.03	P=0.0116	WT vs WT ARU P=0.0472<br>Gpr37 KO vs WT NPD1 P=0.0188<br>WT vs WT ARU P=0.0296
fig7d	ono-way ANOVA	Tukey's multiple comparisons test	F (3, 28) = 5.025	P = 0.0065	Gpr37 KO vs WT ARU P=0.039;<br>Gpr37 KO vs WT NPD1 P=0.0051
fig7f	Log-rank (Mantel-Cox) test	Chi square	4.701	P=0.0301	Gpr37 KO vs WT NPD1 P=0.0301
fig7g	Two-way ANOVA	Tukey's multiple comparisons test	F (3, 78) = 2.914	P=0.0395	Gpr37 KO vs WT NPD1 P=0.0043
fig8c	Two-way ANOVA	Bonferroni's multiple comparisons test	F (5, 48) = 21.97	P<0.0001	WT vs Gpr37 KO P>0.9999
fig8d	Two-way ANOVA	Bonferroni's multiple comparisons test	F (5, 162) = 105.9	P<0.0001	WT vs Gpr37 KO 7d P=0.0016; 10d P=0.0000000000716<br>;14d P=0.0000000000001
fig8e	Two-way ANOVA	Bonferroni's multiple comparisons test	F (5, 48) = 27.98	P<0.0001	WT vs Gpr37 KO 3d P=0.000006
fig8f	Two-way ANOVA	Bonferroni's multiple comparisons test	F (5, 48) = 60.05	P<0.0001	WT vs Gpr37 KO 14d P=0.0028 BL:P=0.0003
fig8h	Two-way ANOVA	Bonferroni's multiple comparisons test	F (4, 80) = 47.32	P<0.0001	WT vs Gpr37 KO 10dP = 0.00001<br>and14d P=0.0000002
fig8i	Two-way ANOVA	Tukey's multiple comparisons test	F (1, 51) = 3.461	P=0.0686	Day1 vs ARU P=0.001
		Bonferroni's multiple comparisons test	F (2, 51) = 367.4	P<0.0001	WT vs Gpr37 KO P=0.0015
sfig1a	Two-way ANOVA	Bonferroni's multiple comparisons test	F (1, 152) = 19.22	P < 0.0001	WT vs Gpr37 KO P=0.0048(14d)
sfig1b	Two-way ANOVA	Bonferroni's multiple comparisons test	F (3, 32) = 0.5312	P=0.6642	WT vs Gpr37 KO P>0.999
sfig1c	Two-way ANOVA	Bonferroni's multiple comparisons test	F (3, 29) = 3.457	P=0.0291	WT vs Gpr37 KO P>0.999
sfig1f	Two-way ANOVA	Bonferroni's multiple comparisons test	F (1, 40) = 0.03565	P=0.8512	
sfig2b	Two-way ANOVA	Bonferroni's multiple comparisons test	F (1, 36) = 12.30	P = 0.0012	WT vs Gpr37 KO P=0.00001
sfig3b	Two-way ANOVA	Tukey's multiple comparisons test	F (2, 40) = 3.501	P=0.0397	Vehicle vs NPD1 P=0.0425
sfig3c	Two-way ANOVA	Tukey's multiple comparisons test	F (2, 34) = 6.633	P=0.0037	Vehicle vs NPD1 P=0.0127<br>Vehicle vs ARU P=0.0071
sfig3d	Two-way ANOVA	Tukey's multiple comparisons test	F (2, 36) = 15.39	P<0.0001	Vehicle vs NPD1 P=0.0003<br>Vehicle vs ARU P=0.000033
sfig3e	Two-way ANOVA	Tukey's multiple comparisons test	F (2, 36) = 3.736	P=0.0336	Vehicle vs NPD1 P=0.00000641<br>Vehicle vs ARU P=0.0014
sfig3f	Two-way ANOVA	Tukey's multiple comparisons test	F (2, 36) = 14.93	P<0.0001	Vehicle vs NPD1 P=0.00000024<br>Vehicle vs ARU P=0.00000033
sfig3g	Two-way ANOVA	Tukey's multiple comparisons test	F (3, 36) = 52.86	P<0.0001	Vehicle vs NPD1 P=0.0293
sfig4b	Unpaired t test	Two-tailed	t=26.92 df=4	P=0.000013	
sfig4c	Unpaired t test	Two-tailed	t=3.980 df=4	P=0.0164	
sfig4e	Two-way ANOVA	Tukey's multiple comparisons test	F (2, 37) = 20.34	P < 0.0001	WT vs WT+ARU P=0.0494
sfig4f	Log-rank (Mantel-Cox) test	Chi square	1.513	P=0.2188	
sfig4g	Log-rank (Mantel-Cox) test	Chi square	0.09854	P=0.7536	
sfig4h	Log-rank (Mantel-Cox) test	Chi square	3.531	P=0.0602	
sfig4i	Two-way ANOVA	Bonferroni's multiple comparisons test	F (2, 37) = 20.34	P < 0.0001	Vehicle vs ARU P=0.0001
sfig4k	Unpaired t test	Two-tailed	t=3.926, df=15	P=0.0013	
sfig5a	One-way ANOVA	Tukey's multiple comparisons test	F (3, 27) = 2.928	P=0.0517	Gpr37 KO vs WT ARU P=0.0063<br>Gpr37 KO vs WT NPD1 P=0.0293
sfig5c	Log-rank (Mantel-Cox) test	Chi square	0.08672	P=0.7684	
sfig5d	Two-way ANOVA	Tukey's multiple comparisons test	F (1, 14) = 3.354	P=0.0884	DMSO vs ARU P >0.9999
sfig6b	Log-rank (Mantel-Cox) test	Chi square	8.581	P=0.0137	WT vs WT+WT P=0.0078<br>WT+WT vs WT+Gpr37 KO P=0.0275
sfig6c	Two-way ANOVA	Tukey's multiple comparisons test	F (3, 101) = 10.32	P < 0.0001	WT vs WT+WT P=0.0015<br>WT+WT vs WT+Gpr37 KO P=0.023
sfig6d	Two-way ANOVA	Tukey's multiple comparisons test	F (1, 21) = 4.036	P = 0.0576	Gpr37 KO WT vs Gpr37 KO Gpr37 KO P=0.0306
sfig6e	Log-rank (Mantel-Cox) test	Chi square	0.6275	P=0.4283	
sfig6f	Two-way ANOVA	Tukey's multiple comparisons test	F (1, 39) = 3.566	P=0.0664	WT+WT vs WT+Gpr37 KO P>0.9999
