# Supplementary material for: Activation of GPR37 in macrophages confers protection against infection-induced sepsis and pain-like behaviour in mice
Source: Nat Commun. 2021 Mar 17;12:1704. doi: 10.1038/s41467-021-21940-8 (PMC7969930; doi:10.1038/s41467-021-21940-8)
Supplement: Supplementary file 3 — Reporting Summary [file 41467_2021_21940_MOESM3_ESM.pdf]

nature research

Corresponding author(s): Ru-Rong Ji

Last updated by author(s): Jan 30, 2021

Reporting Summary

Nature Research wishes to improve the reproducibility of the work that we publish. This form provides structure for consistency and transparency in reporting. For further information on Nature Research policies, see our Editorial Policies and the Editorial Policy Checklist.

Statistics

For all statistical analyses, confirm that the following items are present in the figure legend, table legend, main text, or Methods section.

n/a	Confirmed	
☐	☒	The exact sample size (n) for each experimental group/condition, given as a discrete number and unit of measurement
☐	☒	A statement on whether measurements were taken from distinct samples or whether the same sample was measured repeatedly
☐	☒	The statistical test(s) used AND whether they are one- or two-sided<br>*Only common tests should be described solely by name; describe more complex techniques in the Methods section.*
☐	☒	A description of all covariates tested
☐	☒	A description of any assumptions or corrections, such as tests of normality and adjustment for multiple comparisons
☐	☒	A full description of the statistical parameters including central tendency (e.g. means) or other basic estimates (e.g. regression coefficient) AND variation (e.g. standard deviation) or associated estimates of uncertainty (e.g. confidence intervals)
☐	☒	For null hypothesis testing, the test statistic (e.g. F, t, r) with confidence intervals, effect sizes, degrees of freedom and P value noted<br>*Give P values as exact values whenever suitable.*
☒	☐	For Bayesian analysis, information on the choice of priors and Markov chain Monte Carlo settings
☒	☐	For hierarchical and complex designs, identification of the appropriate level for tests and full reporting of outcomes
☒	☐	Estimates of effect sizes (e.g. Cohen's d, Pearson's r), indicating how they were calculated

Our web collection on statistics for biologists contains articles on many of the points above.

Software and code

Policy information about availability of computer code

Data collection	AutoDock Tools 4, Schrodinger software, GPU-accelerated DESMOND software, BD FACS Diva 8 software.
Data analysis	Image J, Prism GraphPad 6.0. , Cytobank.

For manuscripts utilizing custom algorithms or software that are central to the research but not yet described in published literature, software must be made available to editors and reviewers. We strongly encourage code deposition in a community repository (e.g. GitHub). See the Nature Research guidelines for submitting code & software for further information.

Data

Policy information about availability of data

All manuscripts must include a data availability statement. This statement should provide the following information, where applicable:

- Accession codes, unique identifiers, or web links for publicly available datasets
- A list of figures that have associated raw data
- A description of any restrictions on data availability

All data that support the findings of this study are available from the corresponding author upon reasonable request.

Field-specific reporting

Life sciences study design

All studies must disclose on these points even when the disclosure is negative.

Sample size	The sample size was determined based on our experience with the experimental models, anticipated biological variables, previous literatures.
Data exclusions	No data exclusions were applied.
Replication	The majority of experiments were repeated at least three times to ensure reproducibility. Statistical analyses were done to illustrate significance.The times of replication for each experiment were indicated in figure legend. All attempts at replication were successful.
Randomization	Animals were randomly assigned into different cages at the time of purchase or weaning and randomly assigned into different groups before expeiments.
Blinding	In all of behavior tests, the investigators were blinded to the drug administrations and the groups assignments. All of the drugs were made and injected by another person.

Reporting for specific materials, systems and methods

We require information from authors about some types of materials, experimental systems and methods used in many studies. Here, indicate whether each material, system or method listed is relevant to your study. If you are not sure if a list item applies to your research, read the appropriate section before selecting a response.

Materials & experimental systems

n/a	Involved in the study	
☐	☒	Antibodies
☐	☒	Eukaryotic cell lines
☒	☐	Palaeontology and archaeology
☐	☒	Animals and other organisms
☒	☐	Human research participants
☒	☐	Clinical data
☒	☐	Dual use research of concern

Methods

n/a	Involved in the study	
☒	☐	ChIP-seq
☐	☒	Flow cytometry
☒	☐	MRI-based neuroimaging

Antibodies

Antibodies used

cd16/32, Biolegend, Cat#:101302; F4/80-FITC,Biolegend, Cat#: 123107; CD45 (30-F11), FITC, eBioscience Cat#: 11-0451-82, CD11b-APC, Biolegend, Cat#: 101211, Ly6g APC cy7, Biolegend, Cat#: 127623, anti-V5 antibody, Cell Signaling, Cat#: 80076.
CD11b-PE, Biolegend, Cat#:101207 , CD19-APC, Biolegend, Cat# 115511, CD3-APC cy7, Biolegend, Cat# 100221

Validation

All antibodies utilized for FACS experiments are summarized in Supplementary Table 4.
The antibodies are from commercial sources. For validation, the following methods were used:

1) use of isotype controls for analyses,
2) results from previous publications from our lab,
3) manufacture provided validation on the same species, relevant information on the antibodies are available on the manufacturers' websites.

cd16/32, (https://www.biolegend.com/en-us/products/purified-anti-mouse-cd16-32-antibody-190)
F4/80-FITC(https://www.biolegend.com/en-us/products/fitc-anti-mouse-f4-80-antibody-4067)
CD45 (30-F11), FITC (https://www.thermofisher.com/antibody/product/CD45-Antibody-clone-30-F11-Monoclonal/11-0451-82)
CD11b-APC(https://www.biolegend.com/en-us/products/apc-anti-mouse-human-cd11b-antibody-345)
Ly6g-1 APC cy7(https://www.biolegend.com/en-us/products/apc-cyanine7-anti-mouse-ly-6g-antibody-6755)
CD11b-PE(https://www.biolegend.com/en-us/products/pe-anti-mouse-human-cd11b-antibody-349)
CD19-APC(https://www.biolegend.com/en-us/products/apc-anti-mouse-cd19-antibody-1526)
CD3-APC cy7(https://www.biolegend.com/en-us/products/apc-cyanine7-anti-mouse-cd3-antibody-6068)
anti-V5 antibody(https://www.cellsignal.com/products/primary-antibodies/v5-tag-e9h8o-mouse-mab/80076)

Eukaryotic cell lines

Policy information about cell lines

Cell line source(s)	THP-1 cells (ATCC Cat#TIB-202, from Duke core facility), The HEK293 Flp-In TM cell line (Invitrogen, R78007)
Authentication	THP-1 cells from ATCC were taken from orginial ATCC Cat#TIB-202 stocks. No further authentication procedures were performed.
Mycoplasma contamination	Cells were not mycoplasma positive
Commonly misidentified lines (See ICLAC register)	No commonly misidentified cell lines were used in this study.

Animals and other organisms

Policy information about studies involving animals; ARRIVE guidelines recommended for reporting animal research

Laboratory animals	C57BL/6J (strain 000664) and Gpr37 knockout (strain 005806) mice were purchased from the Jackson Laboratory and maintained at an IACUC-approved Duke University animal facility. Adult male and female mice (8 ~10 weeks) were used to produce infection models and for primary cell cultures for in vitro studies. See Supplementary Table 3 for genotyping primers and Supplementary Table 4 for a complete list of animal sex, genotype, and numbers.
Wild animals	This study did not involve any wild animals.
Field-collected samples	This study did not involve any field collected samples.
Ethics oversight	All the animal procedures were conducted in accordance with the National Institutes of Health Guide for the Care and Use of Laboratory Animals and approved by the Institutional Animal Care & Use Committee (IACUC) of Duke University.

Note that full information on the approval of the study protocol must also be provided in the manuscript.

Flow Cytometry

Plots

Confirm that:

- [x] The axis labels state the marker and fluorochrome used (e.g. CD4-FITC).
- [x] The axis scales are clearly visible. Include numbers along axes only for bottom left plot of group (a 'group' is an analysis of identical markers).
- [x] All plots are contour plots with outliers or pseudocolor plots.
- [x] A numerical value for number of cells or percentage (with statistics) is provided.

Methodology

Sample preparation	1. Peritoneal macrophages (pMΦ) were collected by peritoneal lavage.<br>2. Blood RBC cells were collected by facial vein puncture (30-60 µl).<br>3. THP1 cells were suspended to cultured cells.<br>4. Liver,spleen, and Peritoneal cells were collected after L.m infection or drug applications.
Instrument	BD FACS Diva 8 software (BD Bioscience).
Software	Cytobank (https://www.cytobank.org/cytobank).
Cell population abundance	No sorting was performed. For population abundance in FACS analysis experiments, see specific figures and Supplementary Fig. 1, and Fig 4 and Table 3.
Gating strategy	1. Cells were gated first on FCS-A versus SSC-A to determine all viable cells (G1), FCS-A versus FCS-H to eliminate doublets (G2), and CD45-FITC versus NOS dye negative gating (G3) for analyzing infected RBC levels, or Hoechst 33342 versus FCS-H for detection of phagocyte cells (G3').<br>2. Cells were gated first on FCS-A versus SSC-A to determine all viable cells (G1), FCS-A versus FCS-H to eliminate doublets (G2), and CD11b-PE negative and CD19-APC positive for detection of B cells, cd11b-PE negative and CD3-APC cy7 positive for detection of T cells, F4/80-fitc negative and Ly6g-APC cy7 positive for detection of neutrophils.

- [x] Tick this box to confirm that a figure exemplifying the gating strategy is provided in the Supplementary Information.
